# Feasibility, Acceptability, and Clinical Effectiveness of a Technology-Enabled Cardiac Rehabilitation Platform (Physical Activity Toward Health-I): Randomized Controlled Trial

**DOI:** 10.2196/14221

**Published:** 2020-02-04

**Authors:** Jomme Claes, Véronique Cornelissen, Clare McDermott, Niall Moyna, Nele Pattyn, Nils Cornelis, Anne Gallagher, Ciara McCormack, Helen Newton, Alexandra Gillain, Werner Budts, Kaatje Goetschalckx, Catherine Woods, Kieran Moran, Roselien Buys

**Affiliations:** 1 Physiotherapy Department University Hospitals Leuven Leuven Belgium; 2 Department of Rehabilitation Sciences University of Leuven Leuven Belgium; 3 Department of Health & Human Performance Dublin City University Dublin Ireland; 4 Mater Misericordiae University Hospital Dublin Ireland; 5 Beaumont Hospital Dublin Ireland; 6 Department of Cardiovascular Sciences University of Leuven Leuven Belgium; 7 Physical Activity for Health, Health Research Institute, Department of Physical Education and Sport Sciences University of Limerick Limerick Ireland

**Keywords:** cardiac rehabilitation, physical activity, technology, eHealth

## Abstract

**Background:**

Cardiac rehabilitation (CR) is highly effective as secondary prevention for cardiovascular diseases (CVDs). Uptake of CR remains suboptimal (30% of eligible patients), and long-term adherence to a physically active lifestyle is even lower. Innovative strategies are needed to counteract this phenomenon.

**Objective:**

The Physical Activity Toward Health (PATHway) system was developed to provide a comprehensive, remotely monitored, home-based CR program for CVD patients. The PATHway-I study aimed to investigate its feasibility and clinical efficacy during phase III CR.

**Methods:**

Participants were randomized on a 1:1 basis to the PATHway (PW) intervention group or usual care (UC) control group in a single-blind, multicenter, randomized controlled pilot trial. Outcomes were assessed at completion of phase II CR and 6-month follow-up. The primary outcome was physical activity (PA; Actigraph GT9X link). Secondary outcomes included measures of physical fitness, modifiable cardiovascular risk factors, endothelial function, intima-media thickness of the common carotid artery, and quality of life. System usability and patients’ experiences were evaluated only in PW. A mixed-model analysis of variance with Bonferroni adjustment was used to analyze between-group effects over time. Missing values were handled by means of an intention-to-treat analysis. Statistical significance was set at a 2-sided alpha level of .05. Data are reported as mean (SD).

**Results:**

A convenience sample of 120 CVD patients (mean 61.4 years, SD 13.5 years; 22 women) was included. The PATHway system was deployed in the homes of 60 participants. System use decreased over time and system usability was average with a score of 65.7 (SD 19.7; range 5-100). Moderate-to-vigorous intensity PA increased in PW (PW: 127 [SD 58] min to 141 [SD 69] min, UC: 146 [SD 66] min to 143 [SD 71] min; *P*_interaction_=.04; effect size of 0.42), while diastolic blood pressure (PW: 79 [SD 11] mmHg to 79 [SD 10] mmHg, UC: 78 [SD 9] mmHg to 83 [SD 10] mmHg; *P*_interaction_=.004; effect size of −0.49) and cardiovascular risk score (PW: 15.9% [SD 10.4%] to 15.5% [SD 10.5%], UC: 14.5 [SD 9.7%] to 15.7% [SD 10.9%]; *P*_interaction_=.004; effect size of −0.36) remained constant, but deteriorated in UC.

**Conclusions:**

This pilot study demonstrated the feasibility and acceptability of a technology-enabled, remotely monitored, home-based CR program. Although clinical effectiveness was demonstrated, several challenges were identified that could influence the adoption of PATHway.

**Trial Registration:**

ClinicalTrials.gov NCT02717806; https://clinicaltrials.gov/ct2/show/NCT02717806

**International Registered Report Identifier (IRRID):**

RR2-10.1136/bmjopen-2017-016781

## Introduction

### Background

Globally, cardiovascular disease (CVD) is responsible for over 17.3 million deaths annually and accounts for 45% of noncommunicable deaths [1]. In Europe, over 1.4 million people die prematurely from cardiovascular-related diseases [2] with a projected 25% increase in the incidence of CVD by 2030 [3]. Cardiac rehabilitation (CR) is an important component of the current multidisciplinary approach to the management of patients with various presentations of CVD [2]. Despite the growing evidence of the benefits and importance of CR, uptake remains low with only 30% of eligible patients taking part in an ambulatory center-based CR program and only 50% of those maintaining an active lifestyle 6 months after completion of the program [4].

The reason for low participation rate is multifactorial and includes time constraints, poor accessibility, transportation issues, lack of motivation to change behavior, and low self-efficacy [5]. Home-based programs have proven to be safe [6] and effective [7] and have enormous potential to widen access to CR [8]. Furthermore, home-based CR increases self-efficacy to participate in exercise [9], leading to better adherence to a physically active lifestyle in comparison with usual care groups [10]. However, home-based CR interventions often fail to combine the core components of center-based CR into one intervention [11]. These core components are identified as exercise, education, and psychosocial support [12].

Wearable sensors, often worn as a wristband or embedded in a smartwatch or mobile phone, are now ubiquitous and provide real-time activity and physiological information that allows patients to monitor and adjust physical activity (PA) [13] levels and exercise intensity [14] to meet their rehabilitation goals. Many CVD patients have internet access, use wearable sensors, and have a high interest in technology-enabled home-based CR [15]. In addition, low cost, motion-capturing cameras can facilitate the execution of appropriate movement patterns in the home [16,17]. Frederix et al [11] identified telemonitoring, e-learning, telecoaching, and social networking as the main focus areas of an effective telerehabilitation intervention, but only 16% of publications about telerehabilitation combined 2 focus areas and only 5% combined more than 2 focus areas.

### Objectives

Physical Activity Toward Health (PATHway) was developed as an innovative internet-enabled, personalized exercise platform that incorporates all the core components of CR as well as all focus areas of telerehabilitation [18-20]. It provides regular exercise sessions as the basis upon which to provide a personalized, comprehensive lifestyle intervention program to enable patients to self-manage their CVD and to lead a healthier lifestyle in general. The aim of this trial was to assess the feasibility, acceptance, and short-term clinical effectiveness of the PATHway system for maintaining PA and physical fitness of patients with CVD after completion of an ambulatory center-based CR program.

## Methods

### Study Design

The PATHway-I trial is a single-blind parallel 2-group randomized controlled multicenter pilot study (identifier at ClinicalTrials.gov: NCT02717806) with participant recruitment from 3 European hospitals (University hospitals Leuven [Belgium], Mater Misericordiae University hospital in Dublin [Ireland], and Beaumont University hospital in Dublin [Ireland]), and measurements were performed between May 2017 and July 2018. The study protocol was approved by the ethics committees of UZ Leuven/KU Leuven (Belgium; S59023), the Research Ethics Committees of both Irish hospitals (1/378/1846), and the ethics committee of Dublin City University (DCU; REC2016/123), Ireland. The study adhered to the guidelines set forth by the declaration of Helsinki [21], and participants provided written informed consent before inclusion. The PATHway-I trial was conducted and reported in accordance with the Consolidated Standards of Reporting Trials guidelines [22]. Our full trial protocol has been published previously [23].

### Study Participants

A convenience sample of 120 eligible patients with CVD (aged 40-80 years) was randomized on a 1:1 basis, stratified by country, to usual care control group (UC) or PATHway intervention group (PW) during the last 4 weeks of a phase II outpatient CR program. The randomization schedules were generated for the different centers using a computerized random number generator [23].

The inclusion and exclusion criteria have been reported previously [23]. To be eligible, patients between 40 and 80 years had to have documented CVD for which they were enrolled for the first time in a CR program. Patients needed to be medically and pharmacologically stable and had to have internet access and sufficient space at home for deployment of the PATHway system.

Exclusion criteria were significant illness during the last 6 weeks, known severe ventricular arrhythmia with functional or prognostic significance, significant myocardial ischemia, hemodynamic deterioration or exercise-induced arrhythmia at baseline testing, cardiac disease that limits exercise tolerance (valve disease with significant hemodynamic consequences, hypertrophic cardiomyopathy, etc), comorbidity that may significantly influence 1-year prognosis, functional or mental disability that may limit exercise, acute or chronic inflammatory diseases or malignancy, the use of anti-inflammatory drugs or immune suppression, severe chronic obstructive pulmonary disease (forced expiratory volume in 1 second <50%), New York Heart Association class 4, and participation in another clinical trial.

### Study Interventions

**Figure 1:**
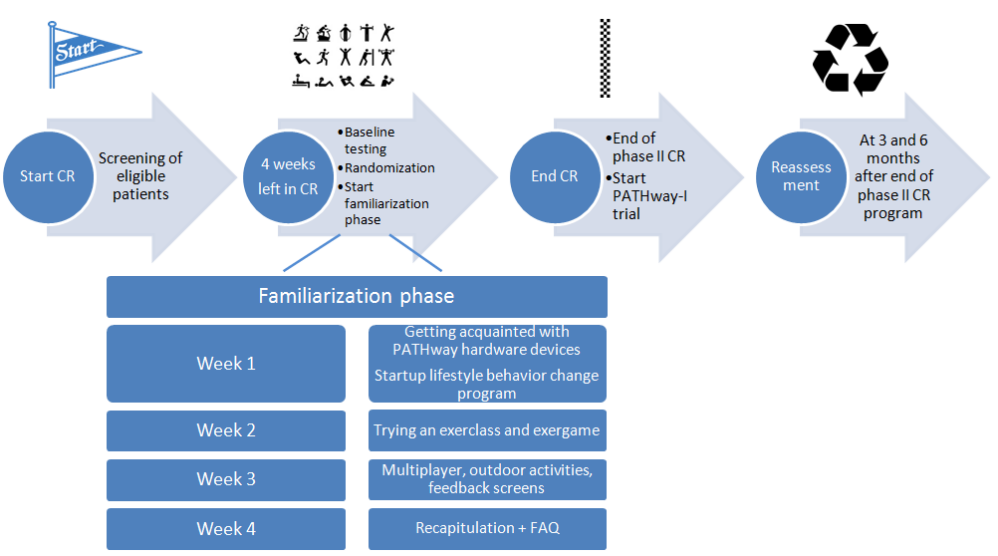
Study flow. CR: cardiac rehabilitation; FAQ: frequently asked questions.

A detailed description of the PATHway system and its development can be found in [18,19,23,24]. During the last 4 weeks of their ambulatory, center-based phase II CR program, participants allocated to PW enrolled in a weekly familiarization session alongside their standard CR to get acquainted with the PATHway intervention [18]. At the same time, the PATHway system was also installed in the participant’s home, and participants were encouraged to interact with the system between familiarization sessions. Figure 1 depicts the flow of participant enrollment in the study and describes the content of each familiarization session. After completion of the center-based CR program, participants completed a cardiopulmonary exercise test (CPET). The results of the CPET were used to determine the individual training heart rates, which were then entered into the PATHway system [25]. Each participant was guided to train at a heart rate between their first and second ventilatory thresholds (VT1 and VT2). Heart rate zones were adjusted according to the results of the 3-month follow-up CPET. Participants were encouraged to achieve the PA goal of 150 min of moderate intensity PA per week according to prevailing guidelines [26]. Different exercise modalities (Exerclass, Exergame, Active lifestyle activity) were available to the participant to achieve this goal [18]. In addition, participants in PW were able to set goals for other lifestyle behaviors, that is, smoking, diet, alcohol consumption, stress, and medication adherence. For each of these goals, the participants had the option to log their behavior and to receive personalized, automatically generated text messages or emails to support adherence and progress toward achieving the goal(s).

Participants allocated to UC received verbal advice on how to best maintain PA and a heart-healthy lifestyle after completion of the center-based CR program [26]. They did not receive direct feedback or support with regard to their PA behavior during the 6-month follow-up period. Both groups continued to receive optimal medical and pharmacological care according to national and international guidelines [27].

### Data Collection and Analysis

Screening procedures and outcome assessments were performed at local study centers (KU Leuven, Belgium, and DCU, Ireland) [23]. PA behavior, physical fitness, vascular function, blood samples, and psychosocial well-being were evaluated 4 weeks before completion of the center-based phase II CR program and reassessed 3 months and 6 months after completion of the center-based CR program. Although staff involved in the intervention delivery and troubleshooting of the PATHway system were clearly not blinded to group allocation, the investigators collecting the outcome measures were blinded to group allocation. Patients were instructed not to reveal their group allocation to these investigators.

### Primary Outcome Measures

The primary outcome was the total amount of PA performed with at least moderate intensity (≥3 metabolic equivalent task units [METs]; moderate-to-vigorous physical activity [MVPA]) per day, measured using an Actigraph GT9X Link (Actigraph). Participants were instructed to wear the Actigraph GT9X Link on the nondominant wrist for 24 h per day during 7 consecutive days. Data collection was considered valid when at least 4 days with a recording period of ≥21 h were available [28]. The Freedson combination algorithm was used to estimate energy expenditure (EE), whereas the Freedson adult algorithm was used to estimate MET and cut points for MVPA [28]. Absolute time spent in sedentary (<0.11 METs), light (0.12-2.99 METs), moderate (3.00-5.99 METs), and vigorous (≥6.00 METs) activity [29], as well as the average amount of steps taken per day, were analyzed.

### Secondary Outcome Measures

#### Health-Related Physical Fitness

Exercise capacity, defined as peak oxygen uptake, was obtained by means of a CPET on a cycle ergometer (Oxycon Pro Jaeger [KU Leuven], Marquette 2000, General electric [DCU]). A 10+10 W/min, 20+20 W/min, or 50+25 W/min protocol was used according to the participants' estimated fitness level to ensure a CPET duration between the recommended 8 min to 12 min [30]. After reaching maximal volitional fatigue, participants cycled for another 3 min at 25 W. A 12-lead electrocardiogram and gas exchange measurements were recorded continuously, and blood pressure was assessed automatically every 2 min (Suntech Tango+, SunTech Medical [KU Leuven], Omron M6-comfort, Omron [DCU]). Peak oxygen consumption was defined as the highest obtained average oxygen consumption over 30 seconds during the CPET [25,31]. The inflection point of the ventilation (VE)/oxygen uptake (VO_2_) ratio and VE/exhaled carbon dioxide ratio graphs were used to determine the VT1 and VT2, respectively [25].

Maximal handgrip strength was measured in both hands using a hand-held dynamometer (JAMAR Dynamometer, Patterson Medical [KU Leuven], TAKEI TKK 5101, TAKEI [DCU]) [32,33], and isometric and isokinetic quadriceps strength and endurance [34] were measured using a Biodex system 3 Pro (Biodex Medical Systems). A 30-second sit-to-stand test was performed according to previously published protocols [35]. The best result of each measure was included in the analysis.

#### Cardiovascular Risk Profile and Vascular Function

Determination of the cardiovascular risk profile included the assessment of body mass index (BMI; body weight/[body length]²), fat percentage, waist and hip circumference, blood pressure and biochemical analysis of blood lipids, plasma glucose, and plasma insulin.

Fat percentage was measured using a bioelectrical impedance device (Omron BF306, Omron [KU Leuven], Tanita BF300, Tanita [DCU]). Waist circumference was measured at the level of the iliac crest and hip circumference at the level of the great trochanter. A minimum of 2 measurements was taken and a third was taken if the initial 2 measurements varied by >1.5 cm [36]. Office blood pressure was measured 3 times at the left upper arm with the participant in fasting state and after a 5-min seated rest (Omron M3, Omron [KU Leuven], Omron M6-comfort, Omron [DCU]) [37]. Blood sampling was performed with the participant in a fasting state and included analysis of plasma glucose, plasma insulin, total cholesterol, high-density lipoprotein cholesterol, calculated low-density lipoprotein cholesterol (LDL-C), and triglycerides. Information relating to current smoking status and the presence/absence of diabetes mellitus was provided by the participant or obtained from their health records. These data were used to calculate the Framingham cardiovascular risk score [38].

High-resolution ultrasonography was used to measure flow-mediated dilatation (FMD) of the right brachial artery (GE Ultrasound Vivid 7, GE Healthcare [KU Leuven], Siemens Acuson, Siemens [DCU]) and carotid intima-media thickness (cIMT; Philips CX-50, Philips [KU Leuven], Siemens Acuson, Siemens [DCU]) of the left and right common carotid arteries. FMD measurements were performed following the protocol of Corretti et al [39]. cIMT measurements used B-mode ultrasound image sequences from the longitudinal section of the common carotid artery, approximately 2 cm below the carotid sinus. For the analysis of both the FMD and cIMT measurements the cardiovascular suite software, developed by Quipu (Quipu srl), was used.

#### Lifestyle, Health Behaviors, and Quality of Life

During each visit, participants completed a series of Web-based surveys on a tablet to assess lifestyle, health behaviors, and quality of life. For an overview of the questionnaires, we refer to the PATHway-I trial protocol [23]. The current report will focus on lifestyle behaviors (smoking, diet, medication adherence, stress, and alcohol consumption) [23], health-related quality of life (HRQoL) assessed by the short-form 36 (SF-36) [40], barriers concerning exercise participation [41], exercise self-efficacy [42], and social support [43] as these are closely related to PA behavior.

#### Feasibility and Usability of the Physical Activity Toward Health System

Adherence to PW was analyzed by generating weekly intervals of the combined upload frequency of Exerclasses, Exergames, and Active Lifestyle activities starting from the familiarization period. Only activities with a total duration between 10 and 500 min were labeled as valid activities and included in the analysis. A distinction was made between all participants in PW and those that actively used the PATHway system. Nonusers were defined as participants randomized to PW without having any valid uploads for Exerclasses, Exergames, or Active Lifestyle activities after the familiarization period, all others were considered active PATHway users.

All participants in PW received a PA goal at setup of the program but were free in choosing whether to receive supporting text messages or emails. On the basis of the scores of lifestyle behavior questionnaires answered at setup of the program, participants in PW were provided with the option to set other lifestyle behavior goals with or without the support of text messages or email. Usage of the behavior change module of the PATHway system [23] was assessed by analyzing the selection of health behavior goals identified by the participant and the number of total messages delivered in support of these goals. Participants who opted to not set other behavior change goals for CVD risk factors or did not want to receive text messages or emails were considered as nonusers of this feature of the PATHway system.

The usability and feasibility of the PATHway system was assessed using the Users Experience Questionnaire (UEQ) [44], System Usability Scale (SUS) [45], and complemented with semistructured interviews guided by the Health IT Usability Evaluation Model [46]. The UEQ provides information relating to each participant's personal impression of the PATHway system. It lists 26 opposing words, for example, not understandable to understandable, separated by a 7-point scale where −3 indicates the most negative answer, 0 indicates a neutral answer, and +3 indicates the most positive answer. An answer below −1 indicates a negative attitude and above +1 a positive attitude toward the product [47]. The words are related to 6 scales: perspicuity, efficiency, dependability, attractiveness, stimulation, and novelty. The first 3 scales indicate the pragmatic quality of a product whereas the latter 2 scales assess the hedonic quality. Attractiveness can be seen as a pure valence dimension [47]. The SUS evaluates the ease of use of the PATHway system by providing 10 items with a 5-point Likert scale, ranging from 1=strongly disagree to 5=strongly agree. For ease of interpretation, the SUS is categorized in 6 levels of usability: *the best imaginable*, *excellent*, *good*, *OK*, *poor*, and *the worst imaginable*. The semistructured interviews were conducted at the end of the 6-month follow-up period and consisted of 2 parts. The first part sought the opinion of the participants' regarding every component of the PATHway system. The second part consisted of 8 open-ended questions. Full details regarding this qualitative research part of the study will be provided separately.

### Safety

A serious adverse event (SAE) was defined as all-cause mortality, hospitalization for CVD, or serious atrial or ventricular arrhythmia. Adverse events (AE) included training-related issues such as muscle, tendon, or joint problems that precluded exercise participation or other diseases that required an interruption of the exercise intervention. All SAE or AE were referred to a data safety and monitoring committee consisting of 4 cardiologists.

### Statistical Analysis

SAS University edition, including SAS studio version 3.71 (SAS Institute Inc) was used to analyze the intervention data. Descriptive continuous data are reported as mean and standard deviation or as median and interquartile range. Categorical variables are reported as observed numbers with percentages. Missing values were handled by means of an intention to treat analysis, which used the *last value carried forward* approach. When baseline data were missing, no imputations were performed. This approach resulted in an uneven number of participants in the analyses.

Potential baseline differences between PW and UC and differences in PA and physical fitness between PATHway users and nonusers were assessed by independent *t* test or Mann-Whitney *U* test where applicable. Categorical variables at baseline and (S)AEs were analyzed using the Chi-square method. Continuous end points were compared between groups by the use of a mixed-effects analysis of variance (ANOVA) using SAS PROC MIXED with the study participant modeled as random effect. The least square mean differences with a Bonferroni adjustment were used to determine differences within groups and between groups. Cohen *d* was used to calculate effect sizes using the averages of the change within the PW and UC.

PATHway usage data were analyzed using RStudio version 3.5.1 (R-foundation). Spearman correlation coefficients were calculated to explore possible links between the PATHway usage and health outcomes and demographics. A correlation of 0.00 to 0.10 was considered negligible, 0.10 to 0.39 as weak, 0.40 to 0.69 as moderate, 0.70 to 0.89 as strong, and 0.90 to 1.0 as very strong [48].

Statistical significance was set at a 2-sided *P* value of <.05.

## Results

### Study Population

**Figure 2:**
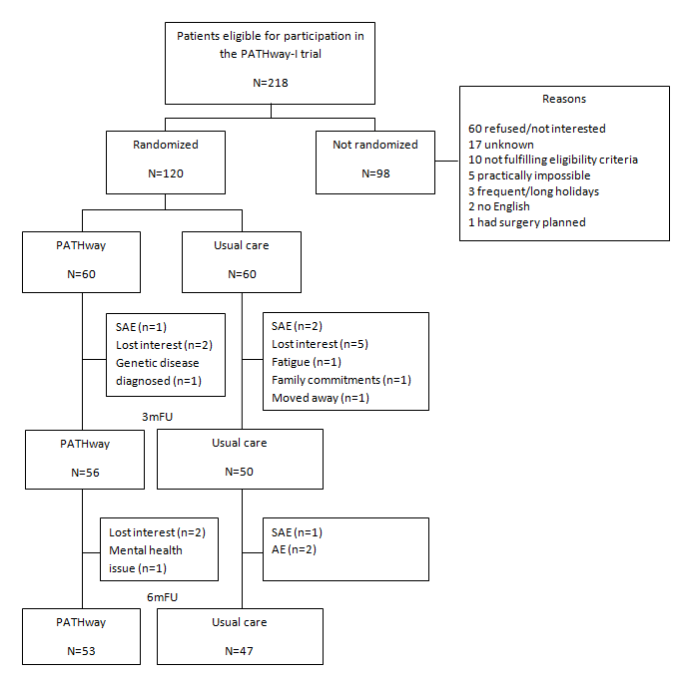
CONSORT (Consolidated Standards of Reporting Trials) flowchart. AE: adverse event; 3mFU: 3-month follow-up; 6mFU: 6-month follow-up; SAE: serious adverse event.

A convenience sample of 120 participants out of 218 eligible patients with CVD was enrolled from May 2017 through December 2017 (Figure 1) at the 3 different sites (University Hospitals Leuven [n=60], Beaumont University Hospital Dublin [n=38], and Mater Misericordiae University Hospital Dublin [n=22]). A total of 20 participants (20/120, 16.7%) dropped out (7 PW, 13 UC) during the 6-month period of which 4 were due to a SAE (Figure 2). Participants who dropped out from the study did not differ from participants who completed the study.

Baseline characteristics of the participants are summarized in Table 1. Except for a higher total EE (*P*=.02) and lower sedentary time in UC (*P*=.045), baseline characteristics of both groups were comparable. This difference in EE remained present also after correcting for bodyweight. The mean age of participants was 61.4 years and 82% were men. A total of 83.3% (100/120) of participants were overweight (BMI>25 kg/m²), and 26.7% (32/120) were obese (BMI>30 kg/m²). A total of 80.8% (97/120) of participants were referred to CR after a percutaneous coronary intervention or coronary artery bypass grafting; and 45.0% (54/120) had a higher degree of education. Upon completion of the phase II CR program, participants showed an average physical fitness level of 96% when compared with their healthy sedentary peers [49]. A comparison between eligible consenting (n=120) and nonconsenting participants (n=98) revealed a significant difference in age, with older participants being less likely to enroll in the study (60.3 [SD 9.2] years vs 64.7 [SD 9.2 years]; *P*=.001).

**Table 1:** Baseline physiological characteristics of the total population and per randomized group.

Baseline characteristics		Total group	PATHway^a^	Usual care
**Descriptive characteristics**				
	Age (n=120; years), mean (SD)	61.4 (13.5)	61.7 (14.5)	59.6 (13.2)
	Gender (n=120; male/female)	98/22	49/11	49/11
	Family history of heart disease (n=119), n (%)	78 (65.0)	37 (61.7)	41 (68.3)
	Atrial fibrillation (n=119), n (%)	8 (6.70)	4 (6.70)	4 (6.70)
	Self-reported diabetes (n=119), n (%)	18 (15.0)	6 (10.0)	12 (20.0)
**Reason for referral**				
	Percutaneous coronary intervention	81 (67.5)	37 (61.7)	44 (73.3)
	Coronary artery bypass graft	16 (13.3)	10 (16.7)	6 (10.0)
	Valve repair	6 (5.0)	2 (3.30)	4 (6.70)
	Other^b^	17 (14.2)	11 (18.30)	6 (10.0)
**Educational level, n (%)**				
	Primary	16 (13.3)	6 (10.0)	10 (16.7)
	Secondary	49 (40.8)	23 (38.3)	26 (43.3)
	Higher^c^	54 (45.0)	30 (50.0)	24 (40.0)
**Physical activity (n=111), mean (SD)**				
	Total daily energy expenditure, kcal/day	1609 (770)	1460 (756)	1754 (575)
	Sedentary time, min/day	700 (120)	716 (154)	691 (104)
	Light physical activity, min/day	585 (106)	575 (115)	588 (104)
	Moderate-to-vigorous physical activity, min/day	127 (101)	124 (70.0)	141 (114)
	Steps, n/day	13,059 (4238)	12,878 (4410)	13,225 (4346)
**Health-related fitness (n=120), mean (SD)**				
	Peak VO_2_^d^, mL/min/kg	23.3 (8.69)	23.2 (8.16)	24.4 (9.84)
	Peak heart rate, bpm	141 (26.3)	142 (24.8)	137 (26.8)
	Wasserman %^e^, mean (SD)	96.0 (27.5)	94.0 (27.5)	96.0 (30.8)
	Peak respiratory exchange ratio	1.25 (0.14)	1.26 (0.14)	1.25 (0.15)
	Borg scale	17.0 (3.00)	17.0 (2.00)	17.0 (4.00)
**Cardiovascular risk profile, mean (SD)**				
	Risk score (n=119), %	12.6 (12.6)	13.6 (15.3)	12.2 (9.70)
	Body mass index (n=120), kg/m²	27.9 (4.54)	27.4 (3.50)	28.2 (5.30)
	Percentage fat (n=120), %	29.2 (8.64)	28.4 (7.41)	30.7 (10.6)
	Waist/hip ratio (n=120)	0.97 (0.08)	0.96 (0.06)	0.96 (0.08)
	Glucose (n=119), mmol/L	5.49 (0.78)	5.50 (0.80)	5.50 (0.70)
	Insulin (N=105), pmol/L	54.4 (44.2)	50.7 (42.1)	56.4 (51.4)
	Total cholesterol (n=120), mmol/L	3.61 (1.18)	3.70 (1.40)	3.50 (1.10)
	High-density lipoprotein cholesterol (n=120), mmol/L	1.22 (0.51)	1.22 (0.53)	1.22 (0.50)
	Low-density lipoprotein cholesterol (n=120), mmol/L	1.75 (0.87)	1.78 (1.02)	1.62 (0.83)
	Triglycerides (n=120), mmol/L	1.03 (0.61)	1.10 (0.64)	0.99 (0.68)

^a^PATHway: Physical Activity Toward Health.

^b^Other includes a combination of coronary artery bypass graft, percutaneous coronary intervention, or valve repair + device implantation.

^c^College or university.

^d^VO_2_: oxygen uptake.

^e^Wasserman %: percent predicted oxygen uptake.

### Primary Outcome: Physical Activity

**Figure 3:**
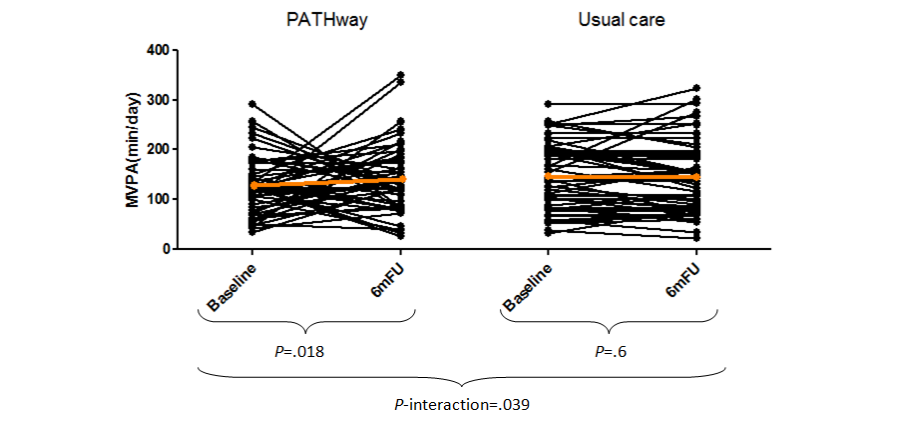
Evolution of moderate-to-vigorous physical activity per day over time in minutes. 6mFU: 6-month follow-up.

Average daily MVPA increased significantly in PW between baseline and 6 months (*P*=.01). There was no change in the average daily minutes of MVPA in UC (*P*=.60; Figure 3). This resulted in a significant interaction effect between groups over time (*P*_interaction_=.04). A significant decrease in low-intensity PA in UC over time was present (*P*=.04), which resulted in a trend toward a significant interaction effect (*P*_interaction_=.11). Table 2 provides point measures on all PA outcomes.

**Table 2:** Intervention effects on daily physical activity behavior (N=111).

Intervention effects on daily physical activity	PATHway^a^, mean (SD)		Usual care, mean (SD)		Effect size	*P* value
	Baseline	6 months	Baseline	6 months		
Total daily energy expenditure, kcal/day	1529 (538)	1560 (563)	1805 (650)	1789 (714)	0.14	.36
Sedentary time, min/day	659 (137)	698 (130)	649 (89.0)	675 (98.0)	−0.32	.11
Light physical activity time, min/day	576 (75)	579 (74)	596 (75.0)	576 (86.0)	0.33	.11
Moderate-to-vigorous physical activity, min/day	127 (57.9)	141 (69.1)	146 (65.9)	143 (70.6)	0.42	.04
Steps, n/day	12,563 (2870)	12,612 (3308)	13,323 (3200)	12,940 (2821)	0.28	.20

^a^PATHway: Physical Activity Toward Health.

### Secondary Outcomes

#### Health-Related Physical Fitness

Health-related physical fitness outcome measures at baseline and 6 months are summarized in Table 3. During the follow-up period, there were no significant differences between groups regarding peak VO_2_ (*P*_interaction_=.64), predicted peak VO_2_ (*P*_interaction_=.79), and VO_2_ at VT1 (*P*_interaction_=.91). Significant time-effects were found for isometric (*P*_time_<.001) and isokinetic (*P*_time_=.046) quadriceps strength as well as the 30-second sit-to-stand test (*P*_time_<.001), without leading to significant interaction effects.

**Table 3:** Intervention effects on health-related physical fitness.

Intervention effects on health-related physical fitness		PATHway^a^, mean (SD)		Usual care, mean (SD)		Effect size	*P* value
		Baseline	6 months	Baseline	6 months		
**Cardiopulmonary exercise testing (n=120)**							
	Peak VO_2_^b^, mL/min/kg	23.8 (5.47)	24.1 (5.82)	24.5 (7.10)	24.5 (6.50)	0.09	.64
	Wasserman %^c^, %	94.0 (21.3)	95.4 (18.5)	98.8 (21.8)	99.3 (20.4)	0.05	.79
	Peak respiratory exchange rate	1.27 (0.11)	1.27 (0.10)	1.24 (0.09)	1.25 (0.10)	−0.11	.55
	VO_2_ at first threshold, mL/min	1106 (314)	1076 (284)	1180 (376)	1155 (339)	−0.02	.91
	Borg score	16 (2)	16 (2)	17 (2)	17 (2)	0.03	.89
**Muscle strength**							
	Handgrip strength dominant side (n=118), kg	40.1 (11.0)	40.1 (11.4)	39.4 (11.0)	39.5 (11.4)	−0.04	.83
	Handgrip strength nondominant side (n=118), kg	38.3 (9.90)	38.7 (10.0)	36.6 (10.1)	36.4 (10.4)	0.11	.57
	Isometric quadriceps strength (n=117), Nm	150 (45.7)	154 (47.1)	149 (48.1)	158 (48.2)	−0.16	.38
	Isokinetic upper leg strength (n=117), J	2085 (725)	2150 (678)	2082 (701)	2124 (677)	0.09	.66
	30s sit-to-stand test (n=118), n	19.0 (4.00)	22.0 (6.00)	18.0 (7.00)	22.0 (7.00)	−0.01	.94

^a^PATHway: Physical Activity Toward Health.

^b^VO_2_: oxygen uptake.

^c^Wasserman %: percent predicted oxygen uptake according to Hansen et al [49].

#### Cardiovascular Risk Profile and Vascular Function

Participants in PW maintained a stable CV risk score, whereas participants in UC increased their risk during the 6-month follow-up period (*P*_interaction_=.03). The same applies for diastolic blood pressure (*P*_interaction_=.004) and the trends that could be seen in waist-hip ratio (*P*_interaction_=.07), LDL-C (*P*_interaction_=.12), and systolic blood pressure (*P*_interaction_=.10; Table 4). There was no significant difference in FMD or cIMT between PW and UC at any time point. There was a significant time main effect for left cIMT (*P*=.03), indicating a significant decrease between baseline and 6 months.

**Table 4:** Intervention effects on cardiovascular risk profile and vascular function.

Intervention effects on cardiovascular risk profile and vascular function			PATHway^a^, mean (SD)				Usual care, mean (SD)				Effect size		*P* value
			Baseline		6 months		Baseline		6 months				
**Cardiovascular risk profile**													
	Risk score (n=115), %	15.9 (10.4)		15.5 (10.5)		14.5 (9.70)		15.7 (10.9)		−0.36		.03	
	Body mass index (n=120), kg/m²	27.4 (3.60)		27.5 (3.60)		28.9 (4.20)		29.2 (4.30)		−0.19		.23	
	Body fat^b^ (n=120), %	28.7 (5.80)		29.2 (5.80)		30.8 (7.30)		31.4 (7.10)		−0.03		.85	
	Waist/hip ratio (n=120)	0.95 (0.09)		0.94 (0.08)		0.96 (0.08)		0.96 (0.08)		0.08		.07	
	Glucose (n=119), mmol/L	5.72 (1.24)		5.83 (1.26)		5.70 (1.66)		5.91 (1.88)		−0.10		.48	
	Insulin (n=104), pmol/L	61.3 (35.3)		71.0 (52.3)		57.1 (29.7)		64.3 (34.9)		0.03		.81	
	HOMA1-IR^c^ (n=104)	2.30 (1.61)		2.73 (2.42)		2.07 (1.11)		2.46 (1.61)		−0.02		.99	
	Total cholesterol (n=120), mmol/L	3.82 (0.97)		3.81 (1.01)		3.66 (0.95)		3.84 (1.08)		−0.25		.15	
	High-density lipoprotein cholesterol (n=120), mmol/L	1.29 (0.36)		1.28 (0.33)		1.30 (0.41)		1.34 (0.47)		−0.18		.29	
	Low-density lipoprotein cholesterol (n=120), mmol/L	1.96 (0.79)		1.95 (0.80)		1.84 (0.78)		2.01 (0.87)		−0.26		.12	
	Triglycerides (n=120), mmol/L	1.25 (0.66)		1.25 (0.65)		1.11 (0.57)		1.20 (0.56)		−0.35		.31	
	Systolic blood pressure (n=120), mmHg	126 (17.0)		127 (16.0)		125 (13.0)		131 (20.0)		−0.27		.10	
	Diastolic blood pressure (n=120), mmHg	79.0 (11.0)		79.0 (10.0)		78.0 (9.00)		83.0 (10.0)		−0.49		.004	
**Vascular function**													
	Brachial artery diameter in rest (n=109), mm	4.17 (0.68)		4.13 (0.62)		4.26 (0.65)		4.33 (0.61)		−0.17		.30	
	Brachial artery diameter post occlusion (n=109), mm	4.49 (0.73)		4.49 (0.66)		4.60 (0.75)		4.63 (0.66)		−0.01		.92	
	Flow-mediated dilatation (n=109), %	8.10 (7.40)		8.90 (4.90)		8.00 (6.40)		6.94 (5.20)		0.28		.20	
	IMT^d^ left (n=114), mm	0.72 (0.15)		0.68 (0.21)		0.71 (0.16)		0.68 (0.15)		0.14		.83	
	IMT right (n=116), mm	0.68 (0.17)		0.67 (0.20)		0.65 (0.16)		0.65 (0.17)		0.14		.66	

^a^PATHway: Physical Activity Toward Health.

^b^Fat%: fat percentage.

^c^HOMA1-IR: Homeostatic Model Assessment of Insulin Resistance.

^d^IMT: intima media thickness.

#### Lifestyle, Health Behaviors, and Quality of Life

Table 5 provides a detailed overview of the questionnaire scores assessing lifestyle, health behaviors, and quality of life. Overall, a small decrease in exercise self-efficacy was observed over the 6-month period (*P*_time_=.03), without differences between groups (*P*_interaction_=.24). Except for a trend toward a subtle decrease in alcohol consumption in PW (*P*_interaction_=.08), lifestyle behaviors including medication adherence, diet, and stress remained stable over time in both groups. Measures of mental well-being, as assessed by the Warwick-Edinburgh Mental Well-Being Scale, were improved after the intervention period (*P*_time_=.03), without any interaction effect between groups. HRQoL as assessed by means of the SF-36 did not change over the 6-month period.

**Table 5:** Intervention effects on lifestyle, health behaviors, and quality-of-life outcomes.

Intervention effects on lifestyle, health behaviors, and quality-of-life			PATHway^a^, mean (SD)				Usual care, mean (SD)				Effect size		*P* value
			Baseline		6 months		Baseline		6 months				
**Mediators of change in physical activity (N=120)**													
	Exercise intentions	21.3 (2.57)		20.5 (3.06)		21.1 (2.67)		20.7 (3.52)		0.14		.48	
	Exercise planning	27.3 (6.30)		28.3 (6.98)		28.4 (5.83)		28.1 (6.73)		0.14		.19	
	Exercise barriers (barriers self-efficacy scale)	68.1 (23.1)		67.3 (22.5)		70.5 (21.5)		68.9 (22.8)		0.04		.83	
	Exercise self-efficacy (exercise self-efficacy scale)	83.3 (17.7)		78.3 (18.3)		81.1 (19.1)		79.7 (20.0)		−0.23		.24	
	Social support (ENRICHD^b^ social support instrument)	27.3 (3.56)		27.2 (3.95)		27.0 (3.36)		27.0 (3.61)		−0.02		.94	
**Lifestyle assessment (N=120)**													
	Diet (Mediterranean diet adherence screener)	6.47 (2.12)		6.35 (1.93)		5.95 (2.08)		6.20 (2.15)		−0.20		.25	
	Medication adherence (Morisky medication adherence scale)	6.78 (0.98)		6.70 (0.89)		6.70 (1.01)		6.78 (0.87)		−0.09		.30	
	Stress (perceived stress scale)	11.4 (7.22)		10.9 (7.84)		11.9 (6.35)		11.6 (6.98)		−0.011		.86	
	Alcohol consumption (alcohol use disorders identification test)	3.23 (2.53)		3.10 (2.43)		2.90 (2.01)		3.08 (2.29)		−0.38		.08	
**Quality of life (N=120)**													
	Health-related quality of life (short form 36)	76.9 (16.5)		77.0 (18.2)		75.0 (16.2)		76.1 (18.3)		−0.11		.60	
	Perceived health (perceived health questionnaire)	3.25 (3.76)		3.43 (4.79)		3.17 (3.34)		3.27 (4.21)		−0.28		.86	
	Mental well-being (Warwick-Edinburgh mental well-being scale)	56.9 (9.42)		57.0 (10.1)		55.0 (8.50)		57.0 (9.65)		0.06		.06	

^a^PATHway: Physical Activity Toward Health.

^b^ENRICHD: Enhancing Recovery in Coronary Heart Disease.

#### Exploratory Analysis of Physical Activity Toward Health Use and Health Outcomes

**Figure 4:**
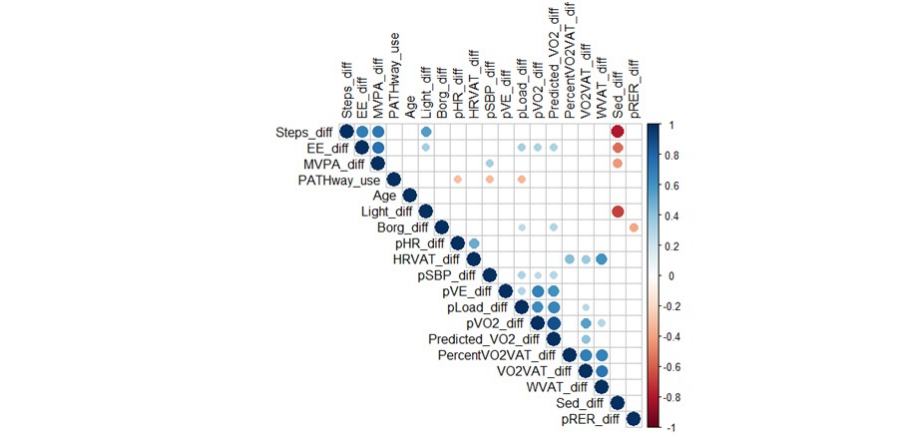
Exploratory analysis of significant correlation coefficients between Physical Activity Toward Health use and demographics, physical activity, and physical fitness. diff: difference; EE: energy expenditure; HRVAT: heart rate at the first ventilatory threshold; MVPA: moderate-to-vigorous physical activity; pHR: peak heart rate; pLoad: peak load; pRER: peak respiratory exchange ratio; pSBP: peak systolic blood pressure; percentVO2VAT: percent oxygen uptake at first ventilatory threshold; predicted_VO2: predicted oxygen uptake according to Hansen; pVE: peak ventilation; pVO2: peak oxygen consumption; VO2VAT: oxygen uptake at first ventilatory threshold; WVAT: load at first ventilatory threshold; Sed: sedentary time.

PATHway use was defined as the total amount of time spent using the Active lifestyle, Exerclass, or Exergame option. If a spearman correlation between PATHway use and age, PA, and physical fitness outcomes was significant, then the magnitude of the correlation is depicted in Figure 4 [49]. Only the change in peak heart rate, change in peak systolic blood pressure, and change in peak load during CPET were significantly correlated with PATHway use, but the correlations were weak with values of −0.30, −0.31, and −0.33, respectively. Furthermore, no significant differences were present concerning PA and physical fitness outcomes between users and nonusers of the PATHway system.

### Feasibility and Usability of the Physical Activity Toward Health System

#### Use of the Physical Activity Toward Health System

**Figure 5:**
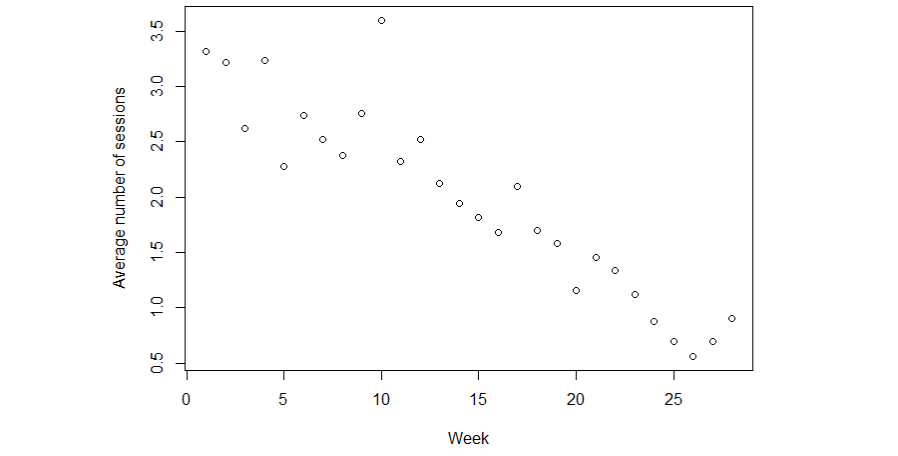
Average amount of exercise sessions per week using the Physical Activity Toward Health system.

The most frequently used PA component was Active lifestyle recorded by means of the Microsoft band 2 (median number of sessions during 6 months: 27, range 2.5-89.5), followed by Exerclasses (median number of sessions during 6 months: 14.5, range: 3-35.8), Exergames (median number of sessions during 6 months: 1, range: 0-3), and manually inserted yet not objectively measured activities (median: 0 range: 0-4). A total of 34 participants (34/60, 57%) set at least one extra goal for CVD risk factor modification using the behavior change module. From the selected goals, 54% related to healthy eating, followed by stress management (17%), alcohol moderation (13%), and medication adherence (12%). PATHway usage decreased over time with a significant lower number of performed exercise sessions using the PATHway system starting at month 4 compared with month 1 (*P*<.001). Figure 5 depicts the decrease in PATHway use over time.

#### Feasibility and Usability

In total, 46 participants (46/60, 77%) in PW completed the UEQ and SUS. Of which, 4 out of 6 scales of the UEQ, including the 2 scales assessing the hedonic quality of a product, had an above average mean score of more than 1 (such as attractiveness, perspicuity, stimulation, and novelty). The 2 other scales (dependability and efficiency), indicating pragmatic quality of a product, scored below average with mean scores of less than 1.

The mean score (SD) of the SUS was 65.5 (19.7), and 5 participants indicated the usability of the PATHway system as *the best imaginable*, 13 participants as *excellent*, 18 participants as *good*, 4 participants as *OK*, 5 participants as *poor,* and 1 participant as *the worst imaginable*.

### Safety

**Figure 6:**
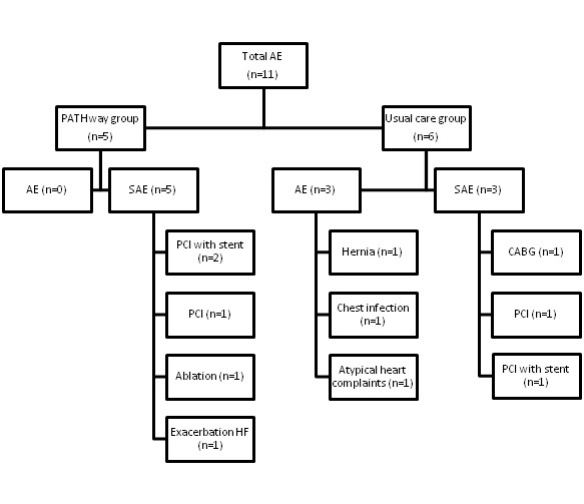
Overview of adverse events during the trial. AE: adverse events; CABG: coronary artery bypass grafting; HF: heart failure; PCI: percutaneous coronary intervention; SAE: serious adverse events.

The rates of AEs were similar in PW and UC (Figure 6). No AEs related to exercise occurred.

## Discussion

### Principal Findings

This study evaluated the feasibility, acceptability, and clinical effectiveness on MVPA and cardiovascular risk profile of a comprehensive technology-enabled, home-based CR system. We found above average scores on 4 out of 6 scales of user experience, 78% of participants indicated at least good usability of the system, and there were no usage-related AEs. Moreover, the PW intervention seems effective for supporting MVPA in daily life after graduating from hospital-based CR.

It was hypothesized that the PATHway platform would aid in maintaining the adherence to a heart healthy active lifestyle following completion of a supervised phase II CR program. MVPA increased in PW by 11%, whereas the levels of MVPA decreased by 2% in UC, resulting in a significant interaction-effect between groups over time and an effect size of 0.42. Previous studies have also reported a better short-term maintenance of PA following telerehabilitation [50,51]. Nevertheless, as absolute MVPA levels at 6-month follow-up are almost equal for both groups in our study, we need to acknowledge that some effects of regression toward the mean might be present, and these positive results need confirmation from larger trials. Our effect on MVPA did not translate into a positive effect on exercise capacity, the parameter most strongly related to morbidity and mortality, indicating that this effect might not be large enough to be clinically meaningful, at least not in this short timeframe of 6 months. Interestingly, daily MVPA at both baseline and 6 months were more than 3 times greater than the values reported by Prince et al [52] regarding CR graduates (63.6 [SD 9.6] years, 75% male, peak VO_2_ after CR of 25.2 [SD 6.6] mL/min/kg) wearing Actigraph GT3X accelerometer at the hip during waking hours. Participants in this study wore the Actigraph GT9X Link on the nondominant wrist for 24 h/day. Research shows that more accurate MVPA results are obtained when the device is worn on the hip compared with the wrist, which might in part be due to the lack of validated accelerometry algorithms for wrist-worn devices [28]. As such, daily MVPA found in this study may have been overestimated. In line with this thought, high MVPA values were also found in a sample of obese individuals wearing the Actigraph GT3X at the wrist [53].

For physical fitness as well as most other outcome measures, we could only document trivial (<.2) effect sizes. Because participants were on average quite fit (96% of predicted sedentary values) at completion of phase II CR, no further improvement in physical fitness was to be expected. Contrary to our hypothesis, whereby we expected a larger decrease in physical fitness in UC, both groups were able to maintain their level of physical fitness. The lack of differences between both groups could be attributable to the small study groups, the motivation to remain fit because of the scheduled follow-up testing as well as the relatively short time period of the trial.

On the other hand, the hypothesized deterioration in UC did occur in relation to the cardiovascular risk score and diastolic blood pressure. Both increased significantly in UC (*P*=.003 and *P*<.001, respectively), while remaining stable in PW and this resulted in significant differences between groups over time. A potential explanation for this finding might be the use of the behavior change module in PW. This module is based on 22 behavior change techniques [18] that can help increase compliance to healthy behaviors and thus have an influence on total cardiovascular risk score [54]. However, our data cannot support this assumption, as the choice to set healthy living goals was not statistically translated into better outcome scores.

Participants’ usage of the PATHway system decreased over time, with the decline starting in the 4th month. Weaknesses of the chosen heart rate tracker, as well as the rather limited suite of exercises/games incorporated into the PATHway system may have been a contributing factor to the decline in usage. Studies examining the use of PA trackers to maintain levels of PA also indicate a gradual decrease in usage, with a sustainability of the use of this technology ranging from 129 days [55] to 5 to 7 months [56]. Factors that increase sustained technology use include ease of use, absence of technology failure, high educational background, younger age, and female gender [55]. It is important to note that use of technology is not necessarily equal to adherence to a desired health behavior. We reported the participants’ engagement with the PATHway system by means of usage data, but engagement is a complex construct and should likely be measured by a combination of methods, which are also context dependent [57].

To maximize engagement, usability, and adherence, the PATHway system was developed in consultation with the target users [18]. The majority of the study participants found PATHway easy to use. However, software problems were identified when the system was first deployed in the participant’s home. The software issues were resolved with 2 major updates during the study, resulting in a more mature system emerging during the later phase of the trial. It is likely that persistent technology-related issues may have frustrated the study participants and have had a negative impact on the use of the system [58]. In line with this thought, the debrief interviews that will be described in detail separately, documented the need for future improvements and expansion to increase the longevity of this mode of CR delivery. In agreement with Hermsen et al [55], we also found that younger CR patients were more likely than older patients to participate in the study. Although almost 90% of participants in PW had completed secondary education and 50% had a higher education degree, we did not find a significant relation between PATHway usage and educational level.

The documented decrease in exercise self-efficacy may also help explain a decrease in use of technology [59]. It is possible that baseline self-efficacy scores are too optimistic because at the time of baseline measurements, the study participants were still participating in supervised, very structured, and well-organized phase II CR. When the participants graduated from CR and needed to implement an exercise routine in their home environment, they may have come to a better understanding of the requirements and challenges of exercise self-efficacy, resulting in lower scores. On the other hand, one might also argue that reaching daily PA goals could also give the participant the feeling he/she no longer needs the PATHway technology to remain physically active [56]. Indeed, the decrease in PATHway usage did not result in a decrease in the daily MVPA and physical fitness.

The study participants in this project were predominantly men, as is in line with how men and women are distributed in ambulatory CR in todays practice in the hospitals participating in this trial. One reason for this might be that the ambulatory CR program in its current format is more appealing to men, compared with women. Furthermore, there is some evidence that women are also significantly less likely to be referred for CR programs following revascularization compared with men [60]. Advances in cardiovascular research is documenting sex-specific differences with regard to diagnosis and treatment of heart disease. To be able to draw conclusions that apply to both sexes, it would have been better to have an equal distribution of women and men in this study. For now, our results should be interpreted with more caution when applied to women with heart disease.

### Limitations

This study was a pilot study including 120 participants. Post hoc power analysis for the outcome measure of MVPA (primary outcome) revealed that our sample size was more than sufficient (100% power). Also, for the cardiovascular risk score, our sample size was sufficient. The number of participants would need to be (much) greater for detecting differences in most of the secondary outcome measures. For example, based on obtained effect size, a sample size of 170 patients in total would have been needed to achieve 80% power at an alpha-error probability of .05 for the outcome measure of peak VO_2_.

Furthermore, it is worth mentioning that a longer follow-up duration might have resulted in a larger difference between intervention and control group because regression to a more sedentary lifestyle might not show straight away in cardiovascular risk factors and physical fitness. Therefore, a longer follow-up should be envisaged in future research with better power.

Because participants could only be enrolled in the study if they completed phase II CR, selection bias toward a highly motivated and physical active study group might have existed. The follow-up period of 6 months can be considered short as the aim of home-based CR is to induce changes in the remaining life of the participant.

Despite extensive testing of the PATHway platform during development, technical errors occurred during the early part of the trial when a significant number of participants started using the PATHway platform at the same time. Complex technology should be stress-tested on a larger scale before implementation in a trial of this size. This seems to be of particular concern for systems that incorporate multiple components, hosted at different sites with use of the internet for communication.

As there are no adequately validated algorithms for the wrist-worn Actigraph GT9X Link, the choice to wear it at the nondominant wrist resulted in high absolute PA values and absolute values might thus not be accurate [28]. However, since we opted for a 24 h/day protocol and the watch had also to be worn during the night, wearing the device at the wrist would most likely result in better wear-time compliance [28], which is why this device was chosen. As both PW and UC received the same wearing instructions and the same set-up protocol was applied at baseline and 6-month follow-up, we believe our results concerning detected differences in PA are reliable.

We observed a significant age difference between consenting and nonconsenting participants, suggesting that caution is warranted when extrapolating the results of acceptability and feasibility of a technological intervention to all CR participants.

### Conclusions

Usage of the PATHway platform for home-based CR following completion of ambulatory CR was demonstrated to be feasible and acceptable for the participants and allowed for safe training sessions. The PATHway platform showed preliminary effectiveness for improving adherence to a physically active lifestyle. The PATHway platform was well received by the users, yet several challenges were identified that should be tackled to result in a more mature technological solution and to increase long-term adoption of a heart-healthy physically active lifestyle. The results of this work can be used as a basis for the design of future RCTs and for sample size calculations to reach statistical power. Future long-term and well-powered studies should focus on implementing those features of the PATHway system that were used most frequently and deemed most useful according to the users. Moreover, the variety of the offered exercises and exercise modes should be increased to improve adherence on the longer term.

## Appendix

Multimedia Appendix 1 CONSORT‐EHEALTH checklist (V 1.6.1).

## Abbreviations

AE: adverse event
ANOVA: analysis of variance
BMI: body mass index
cIMT: carotid intima-media thickness
CPET: cardiopulmonary exercise test
CR: cardiac rehabilitation
CVDs: cardiovascular diseases
DCU: Dublin City University
EE: energy expenditure
FMD: flow mediated dilatation
HRQoL: health-related quality of life
LDL-C: low density lipoprotein cholesterol
MET: metabolic equivalent task unit
MVPA: moderate-to-vigorous physical activity
PA: physical activity
PATHway: Physical Activity Toward Health
PW: PATHway intervention group
SAE: serious adverse event
SF-36: short-form 36
SUS: system usability scale
UC: usual care control group
UEQ: users experience questionnaire
VE: ventilation
VO^2^: oxygen uptake
VT1: first ventilatory threshold
VT2: second ventilatory threshold
